# Recent Advances in the Genetic of MALT Lymphomas

**DOI:** 10.3390/cancers14010176

**Published:** 2021-12-30

**Authors:** Juan José Rodríguez-Sevilla, Antonio Salar

**Affiliations:** 1 Department of Hematology, Hospital del Mar-IMIM, 08003 Barcelona, Spain; 63639@parcdesalutmar.cat; 2 Group of Applied Clinical Research in Hematology, Cancer Research Program-IMIM (Hospital del Mar Medical Research Institute), 08003 Barcelona, Spain; 3 Life and Health Sciences Department, Pompeu Fabra University, 08003 Barcelona, Spain

**Keywords:** MALT lymphoma, marginal zone, *Helicobacter pylori*, extranodal lymphoma, gastric lymphoma

## Abstract

**Simple Summary:**

Mucosa-associated lymphoid tissue (MALT) lymphoma is the most common subtype of marginal zone lymphomas. These B-cell neoplasms may arise from many organs and usually have an indolent behavior. Recurrent chromosomal translocations and cytogenetic alterations are well characterized, some of them being associated to specific sites. Through next-generation sequencing technologies, the mutational landscape of MALT lymphomas has been explored and available data to date show that there are considerable variations in the incidence and spectrum of mutations among MALT lymphoma of different sites. Interestingly, most of these mutations affect several common pathways and some of them are potentially targetable. Gene expression profile and epigenetic studies have also added new information, potentially useful for diagnosis and treatment. This article provides a comprehensive review of the genetic landscape in MALT lymphomas.

**Abstract:**

Mucosa-associated lymphoid tissue (MALT) lymphomas are a diverse group of lymphoid neoplasms with B-cell origin, occurring in adult patients and usually having an indolent clinical behavior. These lymphomas may arise in different anatomic locations, sharing many clinicopathological characteristics, but also having substantial variances in the aetiology and genetic alterations. Chromosomal translocations are recurrent in MALT lymphomas with different prevalence among different sites, being the 4 most common: t(11;18)(q21;q21), t(1;14)(p22;q32), t(14;18)(q32;q21), and t(3;14)(p14.1;q32). Several chromosomal numerical abnormalities have also been described, but probably represent secondary genetic events. The mutational landscape of MALT lymphomas is wide, and the most frequent mutations are: *TNFAIP3*, *CREBBP*, *KMT2C*, *TET2*, *SPEN*, *KMT2D*, *LRP1B*, *PRDM1*, *EP300*, *TNFRSF14*, *NOTCH1/NOTCH2*, and *B2M*, but many other genes may be involved. Similar to chromosomal translocations, certain mutations are enriched in specific lymphoma types. In the same line, variation in immunoglobulin gene usage is recognized among MALT lymphoma of different anatomic locations. In the last decade, several studies have analyzed the role of microRNA, transcriptomics and epigenetic alterations, further improving our knowledge about the pathogenic mechanisms in MALT lymphoma development. All these advances open the possibility of targeted directed treatment and push forward the concept of precision medicine in MALT lymphomas.

## 1. Introduction

Mucosa-associated lymphoid tissue (MALT) lymphoma was first described in 1983 by Isaacson and Wright in the stomach but may arise from other mucosal tissues [1]. According to the WHO classification [2], MALT lymphomas are one of the three recognized subtypes of marginal zone lymphomas (MZL), a group of indolent lymphoid neoplasms which represents 7% of all mature non-Hodgkin lymphomas (NHL) [3]. Based on data from SEER-18 program, MALT lymphomas represent 60.8% of MZLs, followed by nodal MZL (NMZL) (30.3%) and splenic MZL (SMZL) (8.9%) [4]. According to this program, the incidence of MALT lymphomas in the US from 2001–2017 has increased +1.1% per year [4], despite the decrease in the incidence of gastric MALT lymphomas associated with *Helicobacter pylori (H. pylori)* recently described in several studies [5].

MALT lymphomas mainly occur in adults, with a median age about 60 years. Men and women are affected equally, although there is site specific female predominance in the parotid gland and breast [6]. MALT lymphomas can occur at any extranodal site. In health, these tissues are usually almost devoid of lymphoid tissue; however, they accumulate B lymphocytes in response to persistent antigenic stimulation due to chronic infections or autoimmune disorders [7,8]. The most common anatomic sites are the stomach (30%), followed by eye/adnexa (12%), skin (10%), lung (9%) and salivary gland (7%) [4]. However, these lymphomas have been described at many other mucosal organs, such as thyroid, liver, small intestine, large intestine, bladder, dura, and many other sites [7,9].

Gastric MALT lymphoma is linked with chronic *H. pylori* infection, satisfying Koch’s postulates for an etiologic agent [10]. Albeit not with the same clear evidence, other infectious agents have been associated with MALT lymphomas: *Helicobacter heilmannii* in the stomach [11], *Chlamydia psittaci* (*C. psittaci*) in the ocular adnexa [12,13], *Borrelia burgdorferi* in the skin [14,15], *Campylobacter jejuni* in immunoproliferative small intestine disease [16] and, *Achromobacter xylosoxidans* in the lung [17]. Autoimmune conditions have been further associated with MALT lymphoma, including Sjogren’s disease, lymphoepithelial sialadenitis and Hashimoto’s thyroiditis [18]. There are also promising leads related to other infections (hepatitis B and C viruses and human immunodeficiency virus), other B-cell activating autoimmune conditions (systemic lupus erythematosus), trichloroethylene exposure, certain occupations, hair dye, recreational sun exposure, smoking, and alcohol use, but these require further research [4].

MALT lymphomas are considered indolent neoplasms with 5-year relative survival rate of 93.8%, higher than that observed in SMZL (85.3%) and NMZL (82.8%) [4]. Among MALT lymphoma sites, 5-year survival is highest for skin (100%) and lowest for small intestine (87.9%). However, occasionally, MALT lymphomas can progress and transform into aggressive high-grade (diffuse large B cell lymphoma (DLBCL)) and in those cases the survival rate drops sharply.

High throughput genome-wide methodologies, the complete sequencing of the human genome and recent developments in next generation sequencing (NGS) have allowed for unprecedented insights into the genomic alterations that underlie oncogenesis, tumor biology, and survival. Since more than 95% of patients with hematologic malignancies have adequate tissue for genomic profiling, this represents an excellent opportunity to improve diagnosis and classification, to look for prognostic markers and also for detection of pharmacologically tractable targets [19]. This review will summarize the latest advances in the genetics and molecular insights of MALT lymphomas.

## 2. IGHV Usage

A functional B-cell receptor (BCR) is essential for the biology of B-cells. MALT lymphoma cells almost always express surface immunoglobulin (Ig) M and its BCR signalling is functional. This is supported by their proliferative responses to mitogens [20] and the responses achieved with treatment with Bruton Tyrosine Kinase (BTK) inhibitors [21].

MALT lymphomas have highly altered variable heavy chain immunoglobulin (IGHV) and variable light chain immunoglobulin (IGLV) genes, which are consistent with germinal center (GC) or post-GC origin [22,23,24]. A role for antigen-driven clonal expansion of the lymphoma cells is shown through the evidence of ongoing somatic hypermutation in the IGHV [25]. The involvement of antigens is also supported by evidence of clonal evolution within the tumor, suggesting selective pressure to increase affinity of the Ig for antigens [26,27,28]. Despite large mutation loads, the overall structure of the Ig is typically retained in these lymphomas [25].

The variation in Ig gene usage among MALT lymphoma of different anatomic locations is presumably the result of adaptive response and clonal selection induced by their varied aetiologies, resulting in different antigen exposures [29]. The Ig from MALT lymphoma of various anatomic sites is autoreactive rather than recognizing antigens from infectious agents. The auto-reactivity may range from polyreactive to various self-antigens to a high-affinity binding to IgG-Fc, a characteristic of rheumatoid factors (RF) [25,30]. In fact, many of these MALT lymphoma derived immunoglobulins share the fundamental features of known autoantibodies [31]. 

In MALT lymphomas of the salivary gland (SGMZL), there is a clear skewed usage of IGHV1-69/J4 (55%) or IGHV3-7/J3 (15%) rearrangements, and this together with other less frequent (IGHV4-59/J2(J5) and IGHV3-30/JH4) rearrangements indicates that most salivary gland MALT lymphomas express BCR that potentially bears RF activities [32,33].

IGHV4-34 (18%) is the most often usage in ocular adnexal MALT lymphoma (OAMZL), followed by IGHV3-23 (12–17%), IGHV3-30 (10–14%), and IGHV3–7 (9%) [34,35,36,37,38,39]. OAMZL carry no or very rare mutation at the conserved VH FR1 Q6W7A24V25Y26 residues, and in contrast, IGVH4-34 mutations are common at the CDR2 N-link glycosylation site and FR3 K90L91S92 residues [40]. In addition, IGHV gene usage in OAMZL is biased by the presence of *C. psittaci* infection. *C. psittaci*-negative cases have a much greater prevalence of IGHV4-34 usage than those *C. psittaci*-positive [41], raising the possibility that other yet unknown pathogens may be involved in their pathogenesis, boosting the formation of an inflammatory environment in which autoantigen exposure could promote the malignant proliferation of autoreactive cells. Moreover, *TNFAIP3* inactivation by deletion or mutation was significantly higher in MALT lymphomas with IGHV4-34 rearrangement (54%), particularly in those of OAMZL (70%), than in those using other IGHV genes (20%). The concurrence of IGHV4-34 rearrangement and *TNFAIP3* inactivation points toward a cooperative relationship of between these two events in OAMZL lymphomagenesis [37].

MALT lymphomas from other sites have also biased usage of IG genes, albeit those coding for autoantibodies are still overrepresented. Gastric MALT lymphomas (GMZL) have a biased use of IGHV4-34, IGHV3-7 and IGHV1-69 genes [25,30,37,42,43,44,45,46] and those responsive to *H. pylori* antibiotic treatment and without the t(11;18)(q21;q21) of IGHV3-30 or IGHV3-23. MALT lymphomas of the lung and skin have also found to have biased usage of IGHV3 or IGHV4 and IGHVH1-69 or IGHVH4-59, respectively [47]. Moreover, the usage of IGHV4-34 and IGHV1-69 in MALT lymphoma is frequently associated with a biased usage of IGLV (IGKV3-20), suggesting recognition of certain antigenic determinants [25,48]. However, the prognostic impact of both the biased usage of Ig genes or their mutational status (unmutated vs. mutated) remains to be determined in MALT lymphomas.

## 3. Cytogenetics

The identification of cytogenetic abnormalities constitutes an important tool for establishing the diagnosis, monitoring the clinical course, and assessing the prognosis of patients with B-cell lymphomas. Cytogenetic analysis in MALT lymphomas was initially hampered by the facts that biopsies are usually small (most taken by endoscopic procedures) and then rarely subjected to conventional cytogenetic analysis and also, because its proliferation in vitro is often poor. Then, during several years, data were limited in comparison with other types of indolent B-cell lymphomas. However, the application of FISH (fluorescence in-situ hybridization), SKY (spectral karyotyping) and high-resolution technologies such as array based comparative genomic hybridization (array-CGH) have improved our knowledge of chromosomal abnormalities in MALT lymphomas. 

In the last two decades, a variety of chromosomal structural and numerical alterations have been described in MALT lymphomas (Table 1). Chromosomal translocations are recurrent in MALT lymphomas, but not in NMZL or SMZL, and their prevalence differs according to disease sites [49,50]. Many translocations have been reported in MALT lymphomas, but the 4 most common are t(1;14) (p22;q32), t(11;18)(q21;q21), t(14;18)(q32;q21), and t(3;14)(p14.1;q32). All these translocations and their products target the activation of nuclear factor k-light-chain-enhancer of activated B-cells (NF-kB) pathway [51,52,53].

The most common recurrent translocation is t(11;18)(q21;q21) which juxtaposes the N-terminal region API2 gene, containing 3 BIR domains with inhibitor caspase activity, and the C-terminal region of MALT1 gene, containing an intact caspase p20-like domain [54,55,56,57,58,59]. The resulting fusion product undergo oligomerization, generating a chimeric protein that induces aberrant nuclear expression of BCL-10 and activation of both canonical and non-canonical NF-κB pathways, promoting cell survival and proliferation [55,60,61,62,63,64]. A recent molecular mechanism described for the API2/MALT1 fusion protein shows that the tumor suppressor gene LIMA1 binds BIRC2 and is proteolytically cleaved by MALT1 through its paracaspase activity. This cleavage originates a LIM domain-only-containing fragment with oncogenic properties in vitro and in vivo [65].

t(11;18)(q21;q21) is most frequent in MALT lymphomas of the lung (40%) and stomach (20–25%). This translocation is also found in the intestinal (33%; with different frequencies between primary (12.5%) and secondary forms (57%), but rare in colorectal cases), the ocular adnexa (10%), and it is uncommon or not present in MALT lymphomas of the thyroid, salivary gland, and skin [49,50,56,59,63,66,67,68,69,70,71,72]. Aneuploidy is rarely observed. Variant translocations of the t(11;18)(q21;q21) have been described, the three-way translocation t(11;12;18)(q21;q13;q21) in the lung [73,74], t(6;18;11)(q24;q21;q21) in the stomach [75] and t(11;14;18)(q21;q32;q21) in the lung [75].

In the MALT lymphomas of the stomach, t(11;18)(q21;q21) is found in 47% and 68% of cases with stage IE and stage IIE or above, respectively, which do not respond to *H. pylori* antibiotic treatment, but only in 3% of those that respond to *H. pylori* eradication [76,77,78,79,80,81,82,83,84,85,86,87]. Additionally, these t(11;18)(q21;q21) positive cases often show residual disease and have a higher risk of lymphoma relapse after antibiotic treatment [86,88]. Additionally, patients carrying this translocation do also present a poor response to alkylating agents such as cyclophosphamide or chlorambucil [89]. However, treatment with rituximab is active in monotherapy [90] or in combination with chlorambucil [91] or bendamustine [92]. For these reasons, testing for t(11;18)(q21;q21) at diagnosis is commonly recommended to guide treatment choice [93,94]. 

Patients with t(11;18) had more frequent monoclonal gammopathy, in particular of IgM subtype (31% v 8%), some of which developed class switch [86,95]. The link between high IgM and t(11;18) could have come via the CD40 pathway, which induces IgM secretion in B cells [96,97,98]. Finally, this translocation is rarely seen in transformed MALT lymphomas [99].

The t(14;18)(q32;q21) is the second most common among all balanced translocations in MALT lymphoma and brings the *MALT1* gene under the transcriptional control of the IgG enhancer, then MALT1 expression is deregulated fostering NF-kB activation [67,71,100,101,102,103]. MALT1 activation also produces its protease activities, causing specific cleavage and inactivation of NF-κB negative regulators including TNFAIP3 and CYLD, thus further enhancing NF-κB activation [104,105,106,107]. In addition, the lymphoma cells carrying *IGH/MALT1* show not only over-expression of MALT1, but also BCL10 accumulation in cytoplasm. This finding suggests that MALT1 may immobilize BCL10 in cytoplasm through their interaction [102]. Over-expression of MALT1, and also of BCL10, may promote the activation of non-canonical NF-κB pathway via up-regulation of BAFF expression [108]. This rearrangement is seen in 5–20% of MALT lymphomas, especially in the liver (17%), skin (10%), lung (6–9%) and ocular adnexa (7%) [50,67,100,102,109,110]. Other described sites are the salivary gland, dura and kidney among others [111,112,113]. This translocation is also described in rare cases of diffuse large B-cell lymphoma [114,115].

The t(1;14)(p22;q32) juxtaposes the *BCL10* gene under the regulatory control of the *IGH* gene, resulting in deregulated expression [116]. In normal conditions, BCL10 links BCR signalling to the canonical NF-kB pathway, being expressed in the cytoplasm of reactive B-cells [117]. However, BCL10 is aberrantly expressed in the nuclei of lymphoma cells with t(1;14)(p22;q32)/*BCL10-IGH*. Over-expression of BCL10 and formation of CBM signalosome via oligomers uniting the CARD domain results in NF-κB activation [81].

t(1;14)(p22;q32) is particularly associated with MALT lymphomas, albeit it is infrequent (1–2% of MALT lymphomas). This translocation may be seen in MALT lymphoma of the lung (9%) and stomach (4%), but it is rare in other sites: ocular adnexa, salivary gland, thyroid and skin [50,67]. The clinical utility of t(1;14)(p22;q32) is not yet fully characterized. However, retrospective studies suggest that gastric MALT lymphomas with strong BCL10 nuclear expression or t(1;14)(p22;q32) do not respond to *H. pylori* eradication [81,85].

t(3;14)(p14.1;q32), with rearrangement of *IGH* and *FOXP1*, is found in approximately 10% of MALT lymphomas, mainly from cases arising in the thyroid, ocular adnexa and skin. MALT lymphomas from the stomach and the lung, NMZL and SMZL are typically negative. Most MALT patients carrying t(3;14)(p14.1;q32) also harbor additional genetic abnormalities, such as trisomy 3 [118].

Several novel chromosome translocations in occasional cases of MALT lymphoma have been described: t(3;14)(p13;q32)/*FOXP1-IGH* [118,119,120], t(1;14)(p21;q32)/*CNN3-IGH*, t(5;14)(q34;q32)/*ODZ2-IGH*, t(9;14)(p24;q32)/*JMJD2C-IGH* [121], t(X;14)(p11.4;q32)/*GPR34-IGH* [122,123] and t(1;2)(p22;p12) [103,124]. These translocations typically juxtapose the oncogene involved to the *IGH* gene locus, (or with the kappa light chain gene) and cause their over-expression. For instance, FOXP1 over-expression though inhibition of apoptosis and plasma cell differentiation may contribute to the pathogenesis of MALT lymphomas [125,126]. The molecular mechanism causing the oncogenic activities of another aforementioned translocation remains to be investigated. Similar to that seen in the four leading translocations, these lesser common translocations are also restricted to specific sites. For instance, translocation t(3;14)/*FOXP1-IGH* is found in 7–56% of thyroid MALT lymphoma cases, [118,127,128], but is not evidenced in non-malignant thyroid disorders (Hashimoto’s thyroiditis and benign tissue) [127]. Then, detection of t(3;14)/*FOXP1-IGH* may be useful for the differential diagnosis between primary MALT lymphoma of the thyroid and other thyroid disorders [127]. Furthermore, t(X;14)(p11.4;q32) which cause GPR34 over-expression is also restricted to MALT lymphoma of salivary gland, such as MALT lymphomas with *GPR34* mutation [122,123].

Beyond translocations, a spectrum of chromosomal numerical abnormalities has been described in MALT lymphomas. In the first cytogenetic study in MALT lymphomas from the Isaacson’s group, numerical abnormalities of chromosomes 3 and 7 were found in a series of 23 MALT lymphomas [129]. Currently, multiple studies have investigated cytogenetic alterations in MALT lymphomas through several techniques, but in contrast with the aforementioned chromosomal translocations, the role of aneuploidies in lymphomagenesis is less clear and may represent secondary genetic events.

The most common numerical alterations found in MALT lymphomas are trisomy of chromosome 3 or 18, although the frequencies at which these trisomies occur vary markedly with the primary site of disease [130,131]. In cytogenetic studies, trisomy 3 is the most common aberration in MALT lymphomas with a frequency from 20–35% [132,133] to 55–60% [129,131,134], being mainly observed in gastrointestinal, parotid gland and thyroid. By FISH, the prevalence of trisomy 3 is also heterogeneous, from 5–20% [135,136] to 43–85% [131,137,138,139]. These differences could be the result of different primary sites of MALT lymphoma analyzed but also of different technical approaches. Trisomy 3 can co-occurs with trisomy 18 in up to 30% of cases [140] but is mutually exclusive with the translocation t(11;18)(q21;q21). In CGH analysis, partial gain of 3q affecting the regions 3q21-23 and 3q25-29 is reported, indicating that the latter regions are of particular importance and might point to genes involved in the pathogenesis of MZL [141,142].

The genetic mechanisms by which trisomy 3 contributes to lymphomagenesis are not fully clarified. However, the biological effects of chromosomal trisomies are likely to be explained by an enhanced gene dosage effect resulting from larger copy numbers of genes crucial to lymphoma development. Several candidate genes, such as the protooncogene *BCL6* [143], the transcription factor *FOXP1* [143] or the chemokine receptor *CCR4* [144], all found on chromosome 3, have been linked to lymphomagenesis. In addition, trisomy 3 has been recently associated to cause changes at transcriptome levels similar to that seen in the presence of the *BIRC3-MALT1* rearrangement [145]. Of note, patients harboring trisomy 3 were resistant to *H. pylori* eradication treatment in one study [132].

Trisomy 18 has an approximate frequency around 20% [146,147]. Its presence was associated with a tendency to predict recurrence in the stomach [82] and in the ocular adnexa [110]. By CGH analysis, gain of material of chromosome 18 is the second most frequent alteration, and the most common over-represented region can be delineated to bands 18q21-23 [142].

Other trisomies, such as trisomies 7, 12 and others, have been observed non-randomly but less frequently than trisomy 3 or 18. It is worth mentioning that different from other lymphoma types, copy-neutral LOH did not appear to be a common event in MZL [142].

Improvements in the cytogenetic tools have allowed the identification of lesions with relevant role in the lymphomagenesis of MALT lymphomas. Structural aberrations of chromosome 1 frequently involved the chromosomal regions 1p22, 1p34 and 1q21 [129,148] and gains of chromosomes 1q have been associated with progression or lymphoma relapse [141]. Isolated cases of 8q with a common region in 8q22-24 containing *C-MYC* are reported to have rapid disease progression [141]. C-MYC activation via amplification represents a well-known mechanism of disease progression in a wide spectrum of malignant disorders. Loss of material of chromosome 17, especially of 17p, is the most frequent chromosomal loss observed in MZL [141]. Alterations of *TP53* have also been described in a considerable proportion of gastric MALT lymphomas [149,150] and have been associated with high-grade transformation [149].

Other genomic imbalances have also been found using array-CGH [142,151,152]. For instance, deletion of *TNFAIP3* (*A20*) was detected in MALT lymphomas of the ocular adnexa (19%), thyroid (11%), salivary gland (8%), and liver (0.5%), but not in the lung, stomach, and skin [142,153,154,155,156]. In ocular adnexal MALT lymphoma, complete *TNFAIP3* inactivation is associated with reduced lymphoma-free survival [153,157]. TNFAIP3 can inactivate some NF-kB positive regulators including RIP1/2, TRAF6, Ubc13 and NEMO through removing the K63-linked ubiquitin chain, catalyzing the K48-linked polyubiquitination, or direct binding to the linear polyubiquitin chain of its targets [158]. However, *TNFAIP3* inactivation alone is not sufficient for malignant transformation but nonetheless may represent a promising future therapeutic target. In addition, deletion of *CD274* (*PD-L1*) are frequently found in MALT lymphomas of the thyroid, together with mutation in up to 68% of cases (see later) [128].

## 4. Mutations

Although mutational profiling of hematologic neoplasms currently predominates in the area of myeloid neoplasms, there is a growing wealth of literature describing common and highly characteristic genetic alterations in lymphomas. Certain mutations are enriched in specific lymphoma types, and new discoveries continue to emerge at a rapid pace. 

**Figure 1:**
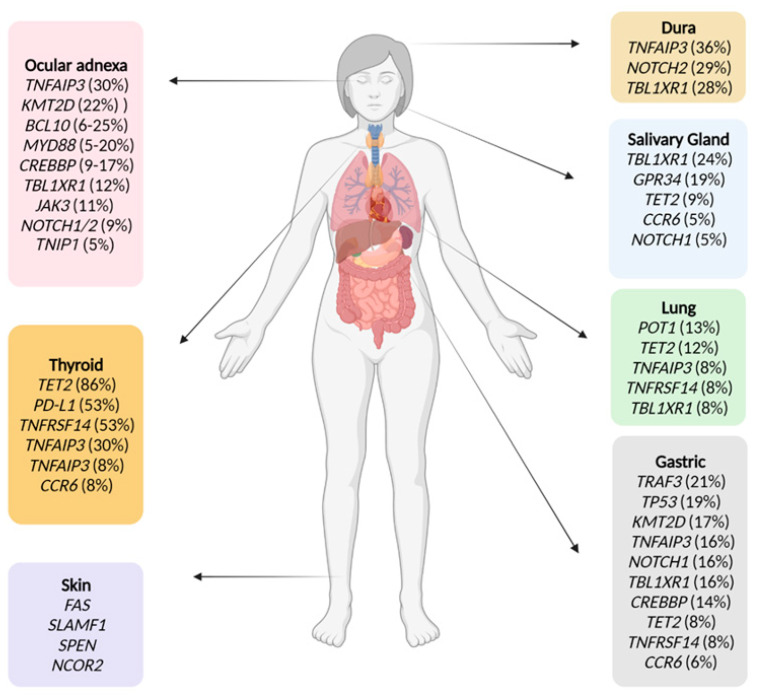
Recurrently mutated genes in MALT lymphoma according to site of origin. Created with BioRender.com.

The mutational landscape of MALT lymphomas is wide and, in the last decade, has been characterized using high-throughput technologies such as whole exome sequencing, whole genome sequencing and customized NGS panels. The data available to date show frequent lesions affecting chromatin remodeling and transcription regulation, BCR and NF-κB signalling, NOTCH pathways and immune surveillance. Not surprisingly, MALT lymphomas show considerable variations in the incidence and spectrum of genetic mutations among different sites, similar to what happens with translocations. Overall, the most frequent mutations are: *TNFAIP3* (29%; including deletions), *CREBBP* (22%), *KMT2C* (19%), *TET2* and *SPEN* (17%, each), *KMT2D*, *LRP1B*, and *PRDM1* (15%, each), *EP300* (13%; includes deletion), *TNFRSF14* (11%; includes deletion), *NOTCH1/NOTCH2* (11%, each) and *B2M* (10%; includes deletion) [145]. Other mutations detected in other studies have been *GPR34* (1–19%) and *CCR6* (1–8%) [159]. Similar to what occurs in follicular lymphoma and DLCBCL, mutations in *KMT2C*, *KMT2D*, *CREBBP* and *EP300* tended to coexist [145] (Figure 1 and Supplementary Table S1).

Some mutations deserve their separate discussion. A study prior the NGS era showed that *TP53* was mutated in 18.8% of gastric MALT lymphoma, and the frequency raised up to 33.3% in those transformed to DLBCL. Only 2% of gastric MALT patients showed the concomitance of *TP53* mutation and allele loss, but 22% of DLBCL displayed both *TP53* mutation and allele loss, suggesting that TP53 partial inactivation might play a role in the development of low-grade MALT lymphomas, whereas complete inactivation might be associated with high-grade transformation [149]. However, in recent studies using NGS technologies, *TP53* has not been usually found to be mutated at presentation in MALT lymphomas from several sites [145,159,160,161].

*MYD88* mutation is infrequent in MALT lymphomas altogether [160,162,163], which is in contrast to primary ocular adnexa MALT lymphoma (OAMZL) where *MYD88* mutation, mainly L265P, occurs at frequencies about 6–7% [159,164,165]. The clinical characteristics are similar in patients with and without *MYD88* mutation, including lesion size, lymphoma stage, recurrence, and response to treatment [163]. This gain-of-function mutation enables MYD88 assembling an active complex containing IRAK1 and IRAK4, triggering signaling cascade to activate NF-κB, STAT3 and AP1 transcription factors [166].

As previously mentioned, *TNFAIP3* (*A20*) inactivation by mutation and/or deletion is frequent in MALT lymphomas, in particular in those arising in the ocular adnexa (29–54%) [145,153,167,168], although it has been described in other locations such as the dura (36%) [169], salivary gland (3%) [159] and thyroid (8%) [159], among others. The vast majority of *TNFAIP3* mutations are deleterious changes (frameshift indels and nonsense mutations), resulting in a truncated protein. Interestingly, *TNFAIP3* mutations rarely co-existed with mutations with other genes, supporting their important pathogenetic role in these lymphomas [170]. *TNFAIP3* truncation mutants show a substantial impairment in repression of NF-kB activation. *TNFAIP3* is a key regulator of inflammation signaling pathways as well as a negative regulator of the NF-κB pathway, which inhibits NF-κB activity triggered by signaling from a variety of surface receptors [171]. Thus, *TNFAIP3* inactivation can increase the activation of the canonical NF-κB pathway triggered by signalling from several receptors including BCR, TLR and TNFR1 due to loss of negative regulation on several signalling molecules (IKKγ, TRAF6 and RIP1/2) downstream of their receptors [172,173,174]. Similarly, *TRAF3* inactivation promotes the activation of the non-canonical NF-κB pathway due to impaired control on NIK degradation [175]. Thus, in gastric MALT lymphoma, alterations of *TRAF3* and *TNFAIP3* were mutually exclusive [176]. Beyond *TNFAIP3* and *MYD88* mutations, MALT lymphoma shows rare or no mutations in other members of NF-kB pathway such as *CD79A*, *CD79B*, *CARD11*, *BIRC3*, and *TNFRSF11A*, which are frequently seen in other B-cell lymphomas with constitutive NF-kB activation. 

NOTCH signalling regulates multiple aspects of lymphoid development and function. Although NOTCH inhibits the earliest stage of B lymphopoiesis, it is nevertheless necessary for the development and maintenance of marginal zone lymphocytes [177,178,179]. However, sustained constitutive NOTCH2 expression alone is insufficient for B-cell lymphomagenesis [180]. Recurrent mutations have been described in *NOTCH1* and *NOTCH2*, but mainly in MALT lymphomas of the ocular adnexa (8–10%) [160] and dura (29%) [169]. These mutations mainly resulted in a shortened *NOTCH1*/*2* product lacking TAD or PEST domains, which governs protein stability and degradation [181]. NOTCH1/2 mutations have been associated to aggressive course in some lymphoproliferative disorders [182,183,184,185,186], but the prognostic impact in MALT lymphomas remains to be determined. In a study of gastric MALT lymphoma, *NOTCH1* mutations were enriched in patients who failed to *H. pylori* eradication [176]. In addition to *NOTCH1*/*2*, genetic lesions in additional NOTCH pathway modulators or members, including *SPEN* (17%) and *DELTEX1,* have been identified [145]. These mutations represent activation modifications that are likely to improve *NOTCH* stability and activity [187].

Several chromatin remodeling and transcriptional regulators are recurrently mutated in MALT lymphoma of different sites, including *TBL1XR1*, *TET2*, *MLL2/KMT2D* and *CREBBP* [37,145,159,160,161,176]. *TBL1XR1* mutations are found in MALT lymphoma of the salivary gland (24%) [159], ocular adnexa (6–18%) [159,160,161,168,188], and in *H. pylori*-resistant gastric MALT lymphoma (16%) [176]. In dural extranodal MZL, recurrent *TBL1XR1* mutations were only seen in association with *NOTCH2* mutations (4/11, 36%), which might indicate a co-operative role for these mutations in lymphomagenesis [169]. The majority of *TBL1XR1* mutations are missense changes affecting regions or residues critical for interaction with NCoR, and may increase TBL1XR1 binding to NCoR and facilitate its degradation, consequently promoting NF-κB and JUN target gene expression [37,159]. *TET2* mutations are frequently found in MALT lymphoma of the thyroid (62%) [128] and will be discussed later. Loss of function *MLL2/KMT2D* mutations have been described in several B-cell lymphomas, and the frequency in MALT lymphomas is variable among studies (5–25%) [145,160,161]. *CREBBP* mutations are also frequent observed (4–22%) [37,145,168,188,189].

Genetic abnormalities that regulate B and T cell interactions have also been described in MALT lymphomas. Mutations in *TNFRSF14* are frequently seen in those arising in the thyroid (46–52%) [128,159] but are also detected in lymphomas from other sites [37,145,159]. Most *TNFRSF14* mutations are deleterious changes such as nonsense, frameshift alterations, thus most likely inactivating or impairing the protein function. Thereby, *TNFRSF14* inactivation may enable malignant B-cells to gain more T-cell help.

As previously discussed, mutations in MALT lymphoma can vary widely depending on the site of origin and, therefore, characteristic locations have been the subject of specific genetic studies. 

OAMZL have been the focus of mutation investigations by several groups. Initial studies found recurrent mutations in *TNFAIP3* (30%), *MYD88* (5–20%), and *BCL10* (6–25%), whereas other genes were rarely or not mutated in the cases analyzed [165,167,190] Later, other studies using whole exome sequencing (WES) or customized NGS panels have found mutations in many other genes, albeit with disparate frequencies. These differences could be attributed to regional variances or different sensibilities in the techniques used that could potentially identify subclonal mutations. Using a NGS panel, Johansson et al., besides validation of mutations previously described, found mutations in *KMT2D* (22%), *NOTCH1* (8%), *NOTCH2* (8%), *TNIP1* (5%), *NFKBIA* (4%), and in other genes at lower frequencies [160]. Using WES, Moody et al., also found a high proportion of patients with *TNFAIP3* mutations (36%), and also recurrent mutations in *MYD88* (7%), *TBL1XR1* (6%), *TNFRSF14* (5%) and *TET2* (4%), besides a few less frequently mutated genes [159]. A further mutation studies identified mutations in additional genes such as *CREBBP* (9–17%) [161,168] and *LRP1B* (6%) [161]. Interestingly, Vela et al., described that up to 65% MALT lymphomas harbored mutations of NF-κB compounds, raising the possibility of precision therapy with BCR inhibitors in refractory cases to conventional treatment. Other genome-wide mutation study identified mutations in *JAK3* (11%) that were located at sites known to alter the protein function and leading to activation of the JAK/STAT signaling pathway by causing a gain of function of *JAK3* [188]. Patients carrying *JAK3* mutation had shorter progression free survival, but not reduced overall survival [188]. Identification of activating *JAK3* mutations in OAML opens the option for potential precision therapeutics by targeting the JAK/STAT pathway with JAK inhibitors. Other newly identified genes recurrently mutated (5–10%) included members of the collagen family (*COL12A1* and *COL1A2*) and *DOCK8* which is involved in RhoGTPase signaling [188]. 

In SGMZL, G-protein coupled receptors have been found mutated at high frequency (*TBL1XR1* = 24%, *GPR34* = 19%) [159]. Interestingly, *GPR34* mutation is almost exclusive of this site and mutations in *GPR34* and *CCR6*, both members of the GPCR family, are mutually exclusive [159].

In MALT lymphomas of the thyroid, Wu et al., have found frequent deleterious mutations of *TET2* (86%), and also mutations in *CD274* (53%), *TNFRSF14* (53%), and *TNFAIP3* (30%) [128]. Both *TNFRSF14* and *TET2* had a significantly higher variant allele frequency than *TNFAIP3*, suggesting that *TNFAIP3* mutations may occur later than *TET2* and *TNFRSF14* changes. In patients carrying *TET2* mutations, 46% of them had two mutations and also had a significantly higher number of somatic variants compared with those without. Most *TET2* mutations are deleterious alterations, inactivating or impairing its dioxygenase activity, hence its role in DNA demethylation, consequently affecting a wide spectrum of gene expression [159,191]. In MALT lymphomas, *TET2* inactivation may deregulate the expression of transcriptional factors indispensable for B-cell function, and thus potentially cooperate with receptor signalling, including those by the enhanced T-helper cell signals, indirectly triggered by *PD-L1/TNFRSF14* inactivation in malignant B-cells [128].

Kiesewetter et al., have recently investigated the genetic characterization of *H. pylori*- negative gastric MALT lymphoma. In addition to reconfirming that the *MALT1* translocation is the most frequent genetic alteration (39%, most likely t(11;18)(q21;q21)/*BIRC3-MALT1*) and *IGH* translocation was further seen in 40% of MALT1-negative cases, mutations in NF-kB signaling pathways were detected in 40% of cases (*TNFAIP3* = 23%, *CARD11* = 9%, *MAP3K14* = 9%). The NF-kB pathway mutations were mutually exclusive from MALT1, albeit not IGH translocation, in total occurring in 86% of cases [189]. Then, *H. pylori*- negative gastric MALT lymphomas harbor activating mutations that affect both canonical and non-canonical NF-kB pathways [192].

Other studies analyzing the mutational landscape of MALT lymphomas at several sites have been conducted and the list of involved genes keeps growing [193].

## 5. MicroRNAs

MicroRNAs are small non-coding RNAs that modulate gene expression on a post-transcriptional level, playing critical roles in cellular proliferation, apoptosis and differentiation [194,195]. MicroRNAs have important roles as tumor suppressor genes and oncogenes in human malignancies, including in lymphomas [196,197]. Only a few studies have analyzed the role of microRNAs in MALT lymphomas, as diagnostic or prognostic tools.

In *H. pylori*-associated gastric MALT lymphoma, Craig et al., identified miR-203 as one of the most strongly downregulated miRNAs in comparison with normal lymphoid tissue, suggesting that might be a key player in the transformation from chronic gastritis to MALT lymphoma. In addition, since ABL1 is the target of miR-203, treatment with imatinib and dasatinib (BCR/ABL inhibitors) prevented tumor cell growth [198]. Zhang et al., have found 53 upregulated and 25 downregulated miRNAs in gastric MALT lymphoma. Subsequently, they found upregulated miR-320a, miR-940, and miR-622, and downregulated miR-331-3p and miR-429 miRNA, and after bioinformatic analysis, miR-320a, miR-622, and miR-429 were found likely to be functionally related to each other as they have the same targets, such as *C-MYC* [199].

miR-142-5p and miR-155 have also been found overexpressed in gastric MALT lymphoma [200]. miR-155, observed during *H. pylori* infection, may be a potential predictor of resistance to *H. pylori* eradication therapy, independently of *API2/MALT1* fusion gene [200]. Inhibition of miRNAs with chemically engineered oligonucleotides, known as “antagomirs”, has been shown to work as specific inhibitors of endogenous miRNAs in mice, and they could be used to silence miR-142 and miR-155 for the treatment of gastric MALT lymphomas resistant to *H. pylori* eradication [201,202].

Our group found through analyses of expression of 384 miRNAs that gastric MALT lymphomas are characterized by specific miRNA expression profile, different to that seen in chronic gastritis and reactive lymphoid tissue. In addition, 17 differentially miRNAs were expressed between patients carrying or not t(11;18)(q21;q21). Overexpression of miR-142-3p and miR-155 and downregulation of miR-203 were detected in gastric MALT lymphomas in comparison with chronic gastritis. These finding suggest that expression levels of these miRNAs might be useful for the differential diagnosis between chronic gastritis and gastric MALT lymphoma [203].

In conjunctival MALT lymphoma, some upregulated miRNAs, such as miR-150/155, as well as downregulated miRNAs, such as miR-184, miR-200a/b/c, and miR-205, have also been identified. Dysregulation of the miR-200 family might be involved in the pathogenesis and progression of the lymphoma [204]. The miRNA-200 family inhibits the initiating step of metastasis, the epithelial-mesenchymal transition, by maintaining the epithelial phenotype through directly targeting the transcriptional repressors [205].

All of these miRNAs may be important in the pathomechanisms of MALT lymphoma. However, further studies are needed to clarify their role in the pathogenesis and also its utility in the clinical practice.

## 6. Transcriptomics

Understanding the transcriptome, the complete set of transcripts in a cell and their quantity, is crucial for interpreting the functional elements of the genome and revealing the molecular constituents of cells and tissues, and also for understanding development and disease [206]. Integration of transcriptome data allows for screening of molecular alterations in deregulated B cells, as well as identification of downstream target genes and pathways, shedding light on the understanding of the lymphomagenesis and in the development of novel therapeutics.

The gene expression profile of MALT lymphomas of the lung was analyzed by Chng et al., who described a prominent T-cell signature and a marginal zone/memory B-cell profile [207]. Gene expression profile also revealed one molecular subset characterized by *MALT1* translocations, having overexpression of NF-KB pathway genes and enrichment for chemokine signaling pathways (CXCR6 and the ligand of CCR5, CCL5). Another subset displayed increased plasma cells and a prominent plasma cell gene signature [207]. In addition, clusters with different biologic characteristics were identified through the analysis of several genes with very high expression in individual samples, such as cases with *MALT1* translocations with high expression of MALT1 and RARA, samples with plasmocytic differentiation having high FKBP11 expression, and cases with high RGS13 expression tending to have trisomy 3 and reactive follicles [207]. CCR4 (3p22.3) was found to be overexpressed in MALT lymphoma bearing trisomy 3 [144].

In 33 MZL cases (18 MALT lymphomas), gains affecting chromosomes 3q and 18 seemed to influence B-cell receptor signaling pathways, cell cycle, Wnt signaling, and apoptosis, whereas genes associated with 3p and 18p gains formed transcripts involved in chemokine and cytokine signaling pathway, ubiquitin proteasome pathway, Ras signaling, and in tight junction regulation [142]. *FOXP1* (3p14.1) is overexpressed in a few MALT lymphomas due to its juxtaposition to the immunoglobulin heavy chain gene promoter after chromosomal translocation [118].

Translocation-positive MALT lymphomas (from all sites) are characterized by an enhanced expression of NF-kB target genes, mainly *TLR6*, chemokine, *CD69, CCR2* and *BCL2*, while translocation-negative cases are characterized by active inflammatory and immune responses, such as interleukin-8, CD86, CD28 and ICOS [208]. The expression of these NF-kB target genes was higher in cases with *MYD88* mutation than in those without the mutation, with TLR6 showing a significant difference [165].

Recently, the transcriptome of MALT lymphomas of the lung harbouring *BIRC3-MALT1* fusion was analysed by Cascione et al. [145], identifying enrichment in MYC target genes, oxidative phosphorylation and DNA repair, genes related to cell cycle, among others. Members of the TNF-receptor superfamily, *TNFSF12-TNFSF13* (*TWE-PRIL*), *TNFRSF17* (*BCMA*), *LTB* (*lymphotoxin β*), and *CXCR3* were among the most upregulated genes in this subset of patients.

In *H. pylori*-positive gastric MALT lymphoma cells, Zou et al., have found 15 pathways differentially enriched, including the Wnt, the mTOR, the NOD-like receptor and the Hippo signalling pathways. By deregulating these pathways, *H. pylori* might influence gastric lymphomagenesis through modulating the proliferation of cells, inducing autophagy and fostering inflammatory responses and epithelial mesenchymal transition. By proteomic analysis, they also identified 116 differentially expressed proteins, most of them previously associated with cancer that can be used as biomarkers for lymphoma diagnosis or as potential therapeutic targets [209].

Zhang et al., conducted a study to identify transcriptomic biomarkers in *H. pylori*-infected gastritis and gastric cancer or MALT lymphoma [210]. In gastric MALT lymphoma, lncRNA GHRLOS and another 44 mRNA were aberrantly expressed, in agreement with previous studies showing upregulation of lncRNA GHRLOS in different solid tumours [211]. In addition, several molecules such as CXCL13, CCL18, CCL19, CCL 20 and TLR 10 were found to be dysregulated in MALT lymphoma, suggesting that not only modulate *H. pylori* infection but also affect the risk of MALT lymphoma [210].

## 7. Epigenetics and Methylation

Epigenetic alterations such as disturbances of DNA methylation and histone modification are common in B-cell lymphomas, contributing to lymphomagenesis [212]. Alterations involving epigenetic regulators have been described in MALT lymphomas from different sites. Inactivation of chromatin remodeling genes are frequently observed in MALT lymphomas, with the most common mutated genes being *TET2, KMT2D, CREBBP, TBL1X1, KMT2C, MT2C, EP300*, among others [37,145,156,157,159,167,169,176] (Figure 1 and Supplemental Table S1). 

In thyroid MALT lymphomas, as previously mentioned, *TET2* mutations were very common with frequencies up to 85.5% [128,159]. Mutations in TET2 has been associated with increased DNA methylation. Interestingly, genes with hypermethylated promoters in TET2 mutated cases were mostly enriched for the PRC2-complex (*EZH2*, *EED* and *SUZ12*) targets, genes bearing histone H3 dimethylation at K4 (H3K4me2) and trimethylation at K27 (H3K27me3). *TET2* mutations have been also described (in percentages below 10%) in the salivary gland, ocular adnexa, stomach, and lung (among others). Since *TET2* mutations are associated with higher response rate to demethylating agents in myeloid leukemias, it is reasonable to investigate the role of demethylating agents in patients with *TET2* mutated MALT lymphomas.

In OAMZL, the most commonly reported epigenetic mutated gene is *KMT2D* with frequencies varying from 14% to 22% [160,161,168], and in *Helicobacter pylori*-negative gastric MALT lymphoma, *KMT2D* (17%) and *CREBBP* (14%) are the two most epigenetic regulator gene mutations [189]. In fact, as previously discussed, mutations in *CREBBP* are found in 4–22% of MALT lymphomas [37,145,168,188,189]. The *CREBBP* gene encodes a lysine acetyltransferase (KAT) protein that activates gene expression through acetylation of histone H3 lysine 18 (H3K18Ac), histone H3 lysine 27 (H3K27Ac), and other residues. KAT domain mutation of *CREBBP* inhibit its catalytic activity and leads to a dominant-repressive effect by preventing the participation of redundant acetyltransferases in transactivation complexes, leading to loss of antagonism to BCL6-mediated gene repression and reduced expression of antigen presentation and interferon signaling genes in germinal-center lymphomas [213]. By using HDAC3-selective inhibitors, these genes can be restored in *CREBBP* mutant cells [214]. This suggests that epigenetic modulation of immune response with HDAC3 inhibitors (or EZH2 inhibitors) may be explored in *CREBBP*-mutant MALT lymphomas, alone or in combination with PD1/PD-L1 blockade (to prevent interferon-induced adaptive immune suppression).

While classical mutations in genes are region-limited, epimutations often occur early in cancer development and have a genome-wide impact. CpG island hypermethylation of *TP15* and *TP73* genes was detected more frequently in gastric MALT lymphomas than in gastric or nodal DLBCL [215]. Hypermethylation of *DAP-k* (72.2%), *GSTP1* (50%), *MGMT* (27.2%) and *TP73* (9%) have been found in MALT lymphomas [216]. Inactivation of the extrinsic pathway of apoptosis through DAP-k methylation, genetic instability favoring acquisition of DNA point mutation caused by MGMT hypermethylation and scavenging reactive oxygen species causing an inflammatory microenvironment by GSTP1 inactivation through promoter methylation may represent pathogenetic events in gastric MALT lymphomagenesis [216]. In MALT lymphomas of the lung, p16 gene methylation was detected at a frequently of 60%, similar to that found in DLBCL (55%), and was not correlated with API2-MALT1 fusion, serum LDH, clinical stage, and increased large cells [217]. The same group showed in MALT lymphoma of the skin that hypermethylation of the CpG islands of the tumor suppressor genes *DAPK* and *TP16^INK4a^* was frequently observed at its initial presentation, but not at tumor progression [72]. These observations suggest that hypermethylation of various genes may be early molecular events that contribute in early MALT lymphomagenesis.

Furthermore, aberrant methylation has also been implicated in tumor progression. Dugge et al., investigated the DNA methylation changes associated with progression of gastric MALT lymphoma through genome-wide DNA methylation profiling and observed that 7698 CpG loci associated with 2419 genes were significantly differentially methylated during gastric MALT lymphoma progression [218]. Among these loci, enrichment of CpGs associated with the promoter was seen and the loci were also enriched in the CpG-rich regions, with most of loci being located within CpG islands [219]. As CpG islands are sites of transcription initiation whose methylation affects chromatin structure, the differentially methylated loci locate to regions involved in transcriptional regulation, which may influence the gene expression. However, no significant changes in the RNA expression levels in the majority of differentially methylated genes were identified. Nevertheless, morphological differences were reflected between gastric MALT lymphoma transformed to large cell lymphoma by characteristic DNA methylation profiles 

Since epigenetic changes are potentially reversible, epigenetic therapy, both alone or in combination with established treatment regimens, has a promising future, leading to potentially less toxic and more effective approaches that standard chemotherapy.

## 8. Applicability in the Real-World

Davis et al., have explored the clinical and practical diagnostic utility of a targeted NGS within mature lymphoid neoplasms (MLN) [220]. They studied the integration of a 40 gene lymphoid panel in 534 MLN during 1-year into the routine comprehensive characterization of lymphomas and found that this practice is not of current diagnostic value in most cases. Improved diagnostic outcomes that led to changing, refining, and facilitating diagnoses were evident in only 5.5% of cases when such testing was applied empirically. Nevertheless, there remains the potential for better outcomes if the practice is standardized [220]. In MZL, from 52 cases, 25 disease-associated variants, 10 variants of uncertain significance, 14 no variant and 3 QLF (qualitative failure due to inadequate DNA quality) were observed. In this study, refinements of diagnoses were most frequently reported due to either the presence or absence of *MYD88* mutations in distinguishing LPL from MZL or the presence of *NOTCH2* and *TNFAIP3* mutations in favoring a diagnosis of MZL over LPL [220].

Pillonel et al., reported a 3-year experience with a 68-gene lymphoma NGS panel applied to 80 cases [221]. The analysis was useful in most cases, helping to confirm or support diagnoses in 35 of 50 histologically difficult cases. However, while it may appear that the diagnostic utility rate is much higher than reported by Davis (70% vs. 5.5%), it should be noted that cases included in the Swiss group were highly selected, based upon a predetermined perceived need for mutational analysis, resulting in only ~1% of all cases being analyzed. NGS analysis was useful to detect potentially therapy or matching drugs were still not available/accessible. However, no patients directly benefited from a matched targeted therapy, which might be mainly linked to the lack of approved agents in lymphoma management. Recently, tazemetostat, a methyltransferase inhibitor, has been approved by FDA for relapsed or refractory follicular lymphoma whose tumors are positive for an *EZH2* mutation and who have received at least 2 prior systemic therapies and also for relapsed or refractory follicular lymphoma who have no suitable alternative treatment options [222]. This precision therapy will change the usefulness of NGS panels for lymphomas in the near future.

Currently, NGS analysis is not generally applied to patients as first-line investigational approach, but rather applicable to further define diagnosis in highly selected otherwise equivocal instances and/or to identify mutations that will guide treatment decisions in cases in which standard treatment options are scarce or suboptimal [221].

## 9. Conclusions

MALT lymphomas are a diverse group of lymphoid neoplasms that exhibit a wide range of genetic features depending on the site of origin. Despite the fact that MALT lymphoma of different anatomic locations shares many common clinicopathological characteristics, there are substantial variances in the aetiology, Ig gene usage, and acquired genetic alterations. Then, it is crucial to better characterize the somatic mutation profile of MALT lymphomas and unravel their molecular oncogenic complexity applying the latest NGS platforms.

Excitingly, the majority of the genetic changes affects NF-KB signal pathway-related genes, resulting in constitutive NF-kB pathway activation. At the same time, since targeted gene sequencing panels have been recently applied to MALT lymphomas, we have learned that other genes are also frequently affected such as those involved in chromatin remodeling, BCR/TLR and NOTCH pathways. The growing knowledge of its genetic landscape, methylation, and transcriptome features raises the possibility of applying new agents to treat a subset of MALT lymphoma patients and also push forward the concept of precision medicine in this type of lymphoma.

## Figures and Tables

**Table 1:** Most common genetic aberrations detected in MALT lymphomas at different sites.

Location	Antigen Exposure Association	IGHV Usage	Abnormality	Involved Genes	Copy Number Variations	Other Imbalances
**GASTRIC**	*Helicobacter pylori* *Helicobacter heilmannii* *Campylobacter jejuni (small intestine)*	IGHV4-34 IGHV3-7 IGHV1-69 IGHV1-2 IGHV3-23	t(11;18)(q21;q21) 20–25% (intestinal 33%) t(1;14)(p22;q32) 4%	*BIRC3-MALT1* *IGHV-BCL10*	Trisomy 3 Trisomy 18	*TNFAIP3* deletion
**OCULAR** **ADNEXA**	*Chlamydia psittaci*	IGHV4-34: 18% IGHV3-23: 12–17% IGHV3-30: 10–14% IGHV3-7: 9%	t(11;18)(q21;q21) 10% t(14;18)(q32;q21) 7% t(3;14)(p14.1;q32)	*BIRC3-MALT1* *IGHV-MALT1* *IGHV-FOXP1*	Trisomy 18 6q gain 30% 3q gain 18q gain	*TNFAIP3* deletion 19%
**THRYOID**	Hashimoto thyroiditis	IGHV3-30	t(3;14)(p14.1;q32) 7–56% t(14;18)(q32;q21)	*IGHV-FOXP1* *IGHV-MALT1*	Trisomy 3	*TNFAIP3* deletion 11% *PD-L1* deletion 53%
**SALIVAL GLAND**	Lymphoepithelial sialadenitis Sjögren syndrome	IGHV1-69/J4: 55% IGHV3-7/J3 15% IGHV4-59/J2(J5) IGHV3-30/JH4	t(X;14)(p11.4;q32)	*IGHV-GPR34*		*TNFAIP3* deletion 8%
**SKIN**	*Borrelia burgdorferi*	IGHV1-69 IGHV4-59 IGHV3-30	t(14;18)(q32;q21) 10% t(3;14)(p14.1;q32),	*IGHV-MALT1*		
**LUNG**	*Achromobacter xylosoxidans*	IGHV3 IGHV4-34	t(11;18)(q21;q21) 40% t(11;12;18)(q21;q13;q21) t(11;14;18)(q21;q32;q21) t(1;14)(p22;q32) 9% t(14;18)(q32;q21) 6–9%	*BIRC3-MALT1* *IGHV-BCL10* *IGHV-MALT1*	3q gain 18q gain	

## Supplementary Materials

The following are available online at https://www.mdpi.com/article/10.3390/cancers14010176/s1, Table S1. Recurrently mutated genes in MALT lymphomas.
