# Supplementary material for: Recent Advances in the Genetic of MALT Lymphomas
Source: Cancers (Basel). 2021 Dec 30;14(1):176. doi: 10.3390/cancers14010176 (PMC8750177; doi:10.3390/cancers14010176)
Supplement: Supplementary file 1 [file cancers-14-00176-s001.zip › cancers-1463122-supplementary.pdf]

Supplemental Table S1. Recurrently mutated genes in MALT lymphomas. Review of references with more than 10 patients and testing more than 5 genes. Include series with MALT lymphoma at different sites and also series with specific locations.

	**Bi, 2012 Yan, 2013**	**Johansson, 2016**	**Gonapathi, 2016**	**Cani, 2016**	**Jung, 2017**	**Moody, 2017**	**Moody, 2018**	**Hyeon, 2018**	**Cascione, 2019**	**Johanson, 2020**	**Vela, 2020**	**Wu, 2021**	**Kissewetter, 2021**
**N**	105	63	14	21	10	179	115/58/13/36	19	72	13+82	34	76	35
**Sites**	OAMZL	OAMZL	Dura	OAMZL	OAMZL	All	OAMZL/ SGMZL/ Thyroid/Stomach	Stomach, resistant	All	OAMZL	OAMZL	Thyroid	Stomach, HP negative
**Cell cycle**													
**BTG1**											6		
**DNA-damage**													
**NSH3**								11					
**NLH1**								11					
**BRCA1**								11					
**BRCA2**								11					
**BRIP1**								11					
**TP53**								5			6		6
**RB1**								5					
**ATM**													6
**NOTCH**													
**NOTCH1**		8			6		2/5/0/11	16	11*				11
**NOTCH2**		8	29						11*				6
**SPEN**									17				
**RAS/MAPK/ERK**													
**MAP3K7**								5					
**MAP3K14**													9
**NF1**								16					
**Immune escape**													
**B2MG**									10				
**PDL1 (CD271)**												52.6	
**Transcription factors**													
**POU2F2**								5					
**CIITA**								16					

**FOXO1**											6		
**POU2AF1**											6		
**ZFHX3**													
**PRDM1**									15				
**KLF2**												12	
**JAK2**								16					
**JAK3**								5		11			
**STAT5B**								5					
**Chromatin modifiers**													
**TET2**							4/9/62/8	5	17			85.5	9
**ARID1A**								5					9
**TBL1XR1**			29		18		6/24/0/8	16		14	18	8	
**KDM5A**								11					
**JARID2**								11					
**EWSR1**								11					
**WHSC1**					6			11					
**KMT2D**		22	14		17	3					18	14	17
**CREBBP**						4			22	13	9	7	14
**EP300**									13		6		
**KMT2C**									19				
**RNA splicing/metabolism**													
**SETBP1**								11					
**REV3L**													6
**NFkB**													
**TRAF3**								21					
**TNFAIP3(A20)**	28.6	27	36		54	19	36/3/8/14	16	29		29	30	23
**CD36**								16					
**NFKBIA**		4						5					
**BCL10**		6			4						9		
**TNIP1**		5											
**BRC/TOLL**													
**CARD11**	0				4			5					9
**MYD88**	5.7	19		25	4	3	7/2/0/0				9		
**TNFRSF14**						3	5/3/46/8		11			52.6	
**Apoptosis**													

**BIRC3**	3												
**WNT**													
**APC**								11					
**Others**													
**PI3K3CD**							3/9/23/0						
**KLHL6**			14										
**CXCR5**												9	9
**CCR5**													6
**CXCR3**												7	
**CCR6**							1/5/8/6					4	
**GPR34**							1/19/0/0						
**CAD**								11					
**ECT2L**								11					
**IGLL5**											9		
**PTPN14**											6		
**COL12A1**	7												
**COAL1A2**	6												
**DOCK8**	6												
**ADAMTS13**	6												
**KRT6B**	6												
**CELSR1**	5												
**RYR1**	5												
**LPRB1**					6				15				
**BRD4**					6								

OAMZL: ocular adnexa marginal zone lymphoma; SGMZL: salivary gland marginal zone lymphoma; HP: *Helicobacter pylori*; * 11% both genes.
